# MR-compatible antibiotic interlocked nail fabrication for the management of long bone infections: first case report of a new technique

**DOI:** 10.1186/1754-9493-8-14

**Published:** 2014-03-17

**Authors:** Cyril Mauffrey, George W Chaus, Nathan Butler, Heather Young

**Affiliations:** 1 Denver Health Medical Center, Department of Orthopaedic Surgery, 777 Bannock Street, Denver 80204, Colorado, USA; 2 Denver Health Medical Center, Department of Infectious disease, Denver, Colorado, USA

**Keywords:** Antibiotic nails, Cement nails, Long bone osteomyelitis, Fabrication of antibiotic nails

## Abstract

Successful management of intramedullary long bone osteomyelitis remains a challenge for both surgeons and patients. Patients are often immune-compromised and have endured multiple surgeries. Treatment principles include antibiotic administration (systemically +/- locally), surgical debridement of the infection site and stabilization. Since their description in 2002, antibiotic coated nails have become part of the armamentarium for the treatment of osteomyelitis allowing both local elution of antibiotics and stabilization of a debrided long bone. Limitations to their utilization have remained, in part from the technical difficulty of fabrication and MRI artifacts. We describe a new surgical technique of fabrication that has the advantages of being simple, reproducible, with an end product free of MRI artifacts.

## Introduction

Successful management of intramedullary long bone osteomyelitis remains a challenge for surgeon and patient alike. Patients are often immune-compromised and may have endured multiple surgeries. Treatment principles initially described by Cierny in 1983 include antibiotic administration (systemically +/- locally), surgical debridement of the infection site and stabilization [1]. The Cierny classification includes an anatomic description of the infection and a description of the host’s resistance to infection [2]. The anatomic description is divided into four types (I-medullary, II-superficial, III-localized full-thickness, IV-diffuse). The description of host’s resistance is divided into three classes (A-healthy, B-locally or systemically impaired, C-major systemic disorder). Since their description in 2002 [3], antibiotic coated nails have become part of the armamentarium for the treatment of osteomyelitis allowing both local elution of antibiotics and stabilization of a debrided long bone [3-14]. Limitations to their utilization have remained due to the technical difficulty of their fabrication and presence of MRI artifacts. There is great variation in the techniques utilized to fabricate antibiotic nails. A guide wire, Enders rod or more recently an interlocked nail, provide stability to the construct. Utilization of interlocked nail has shown satisfactory results while allowing for rotational stability [12,14]. A chest tube is often the best mold available to fabricate the cement nail. Cement nail extraction from the tubing system remains a frustrating exercise, complicated by the melting of plastic and subsequent adhesion to the cement. The technique that we describe is a rapid and reproducible exercise. In addition the utilization of a radiolucent carbon fiber interlocked nail (Carbo-Fix, Champlain, IL, U.S.A) allows for a solid, rotationally stable, MRI compatible fixation. Where inflammatory markers are not always reliable, this latter characteristic enables MRI monitoring of infection with an intramedullary implant in place.

### Indications

The indication for antibiotic nailing of infected long bones should be made on a case-by-case basis. Advantages of this treatment method include early weight bearing, local antibiotic elution and stability. In addition, patients who refuse or are not candidate for long-term external fixation may benefit from antibiotic nailing. Caution is required for well-localized active infection as the reaming process may spread the infected tissues proximal and/or distal into the medullary cavity or neighboring joints.

**Figure 1:**
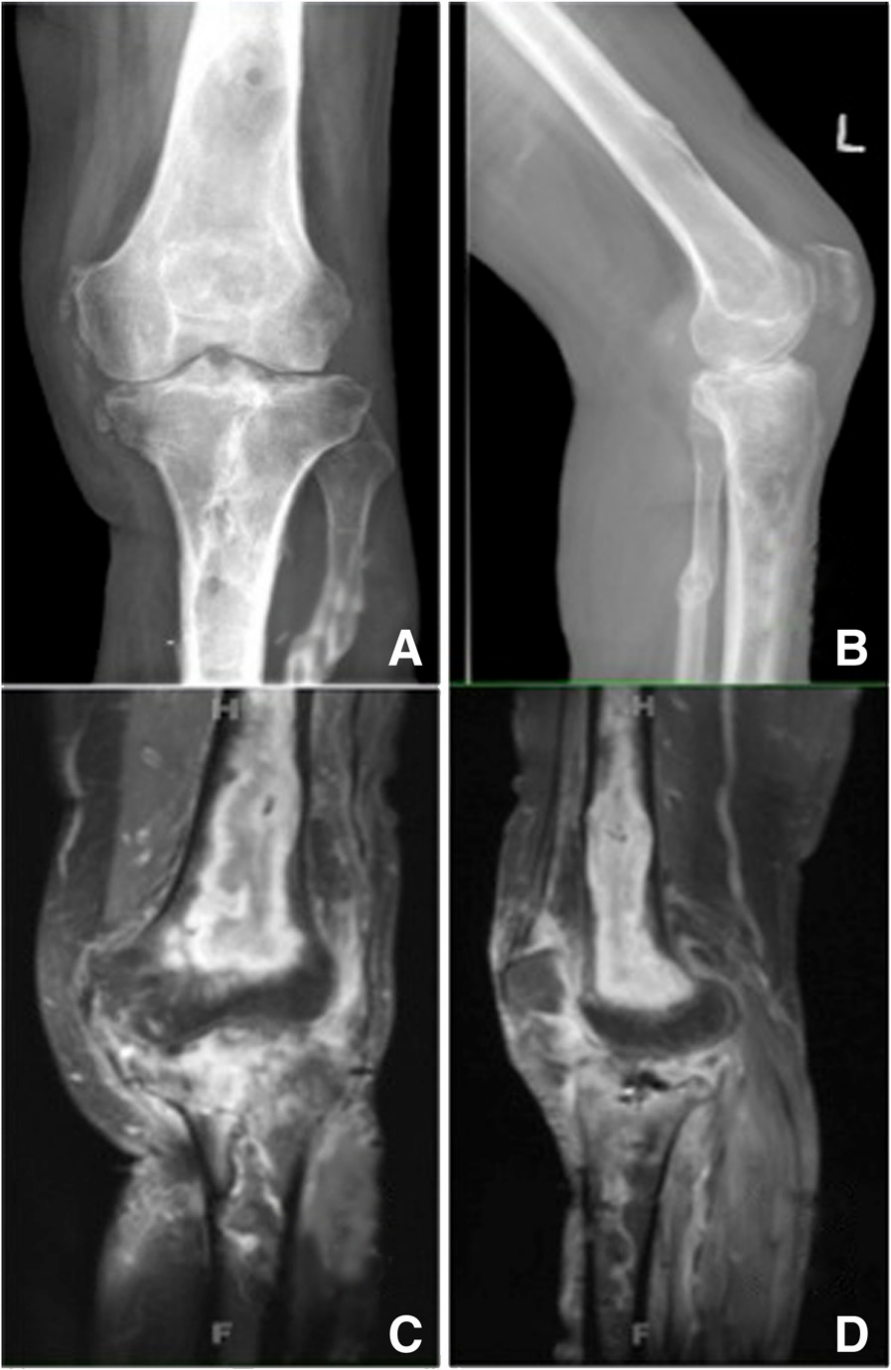
**Preoperative radiographs and MRI scans of the affected femur.** Pre-Operative Images: Anterio-posterior **(A)** and lateral **(B)** X-ray of a left leg revealing a large lytic lesion of the distal femur. In **(C)** and **(D)** coronal and sagittal STIR MRI sequences revealing the distal femoral osteolmyelitis.

We illustrate our technique with the case of a 58-year-old male who presented to our level I trauma center with a new onset of pain with ambulation and swelling around his knee. The patient had a remote history of an ipsilateral proximal tibia and fibula chronic osteomyelitis (trauma related). Six months prior, the patient had been treated for an ipsilateral native knee septic arthritis. Past medical history included diabetes mellitus and hepatitis C making him a class B host according to Cierny and Mader classification [2]. His clinical exam revealed the absence of draining sinus and well healed proximal tibial wounds. No erythema noted around the thigh but tenderness to palpation of his distal femur. A restricted knee range of motion from -15 to 90 degrees was noted as a sequel of the septic arthritis. His imaging studies are shown in Figure 1. An x-ray of his knee revealed a large distal femoral lytic lesion and follow up MRI scan showed diffuse uptake and a pattern consistent with intramedullary osteomyelitis. The patient has type I osteomyelitis according to the anatomic classification of Cierny and Mader [2]. To rule out malignancy, a surgical biopsy was undertaken confirming a diagnosis of chronic osteomyelitis.

Given the extent of the infection, the involvement of the tibia, prior diagnosis of knee joint septic arthritis and a significant risk of pathologic fracture, we elected to treat the patient with closed surgical debridement and stabilization. The risk of pathologic fracture can be estimated based on the scoring system published by Mirel [15]. In this scoring system, points from 1 to 3 are assigned for four categories (location, size of the lesion, pain, and appearance of the lesion). In our case, a score of 9 was assigned (lower extremity 2 points, moderate pain 2 points, lytic lesion 3 points, and size 2 points). Scores >8 points are recommended for prophylactic fixation. An antibiotic intramedullary interlocked carbon fiber nail was selected to provide stability, allow elution of antibiotics and monitoring of treatment response via MRI with limited artifact. A Reamer Irrigator Aspirator (RIA) (Depuy Synthes, Paoli, PA, U.S.A) was utilized to debride the intra-osseous lesion and prevent proximal extension of the infected tissue.

### Surgical technique and fabrication

The patient is placed supine on a radiolucent table with a radiolucent bump placed under the operative extremity. The approach and technique for retrograde femoral nailing is well described in the literature [16]. We use the RIA to ream and debride the intramedullary canal preventing propagation of the infected tissue proximally in the femoral shaft. Reamings should be sent to pathology and microbiology for confirmatory diagnosis. A carbon fiber intramedullary nail (Carbo-Fix, Champlain, IL, U.S.A) with a 8.5 mm diameter is selected. Carbon fiber characteristics include MRI compatibility for monitoring of the infection. The choice of a humeral nail instead of a femoral nail allows for a thinner diameter and therefore a thicker mantle of antibiotic loaded cement.

**Figure 2:**
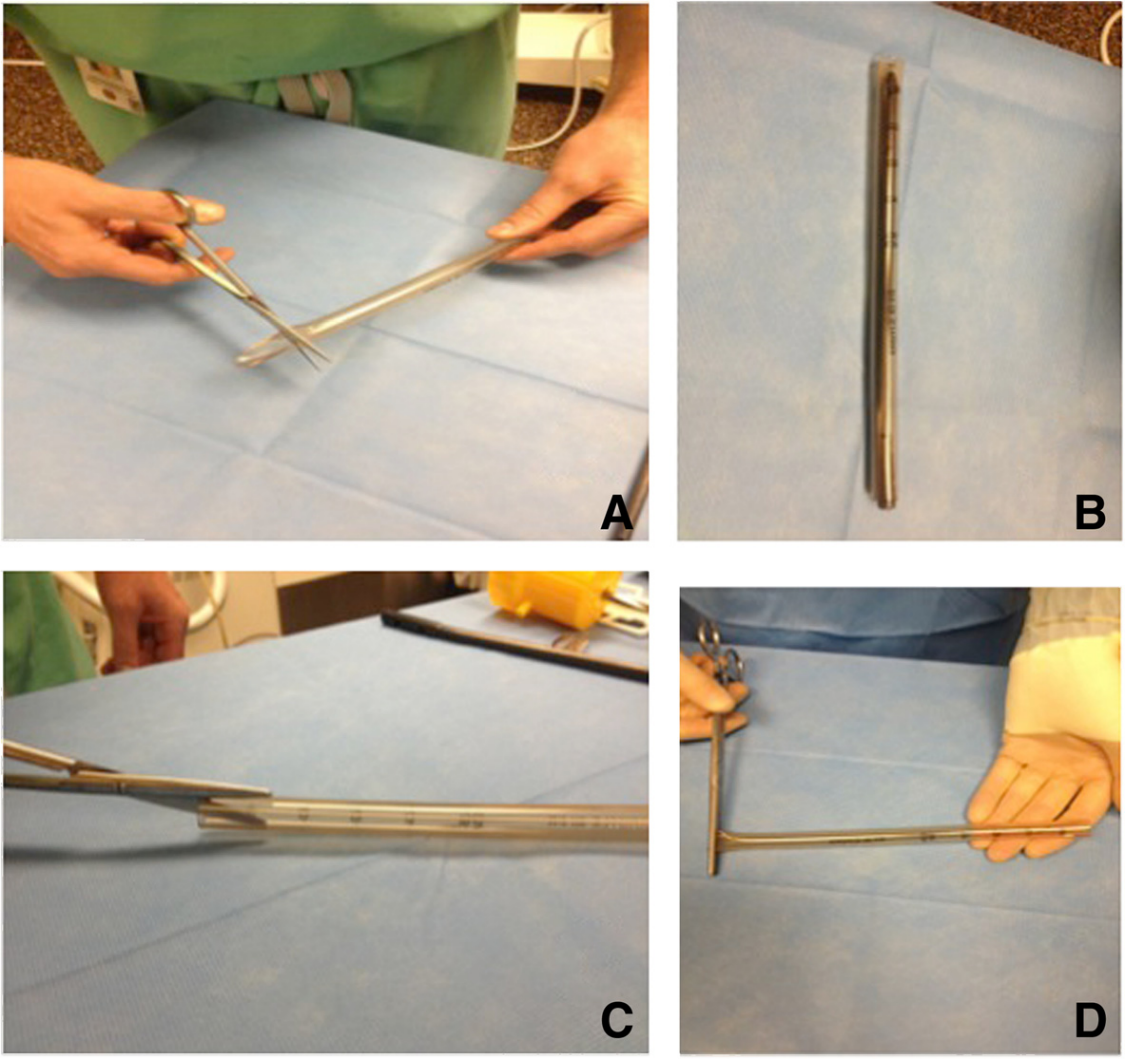
**Initial steps for the preparation of the plastic mold.** Preparation: In preparation for making the antibiotic loaded nail, a 40 Fr chest tube is cut to length **(A)** making sure to leave the proximal end room **(B)** for the aiming arm attachment. Two 2 cm cuts are made on opposing sides of the proximal end of the chest tube **(C)** to facilitate its removal. Using a pair of Kocher, the distal end of the chest tube mold is closed off **(D)**.

**Figure 3:**
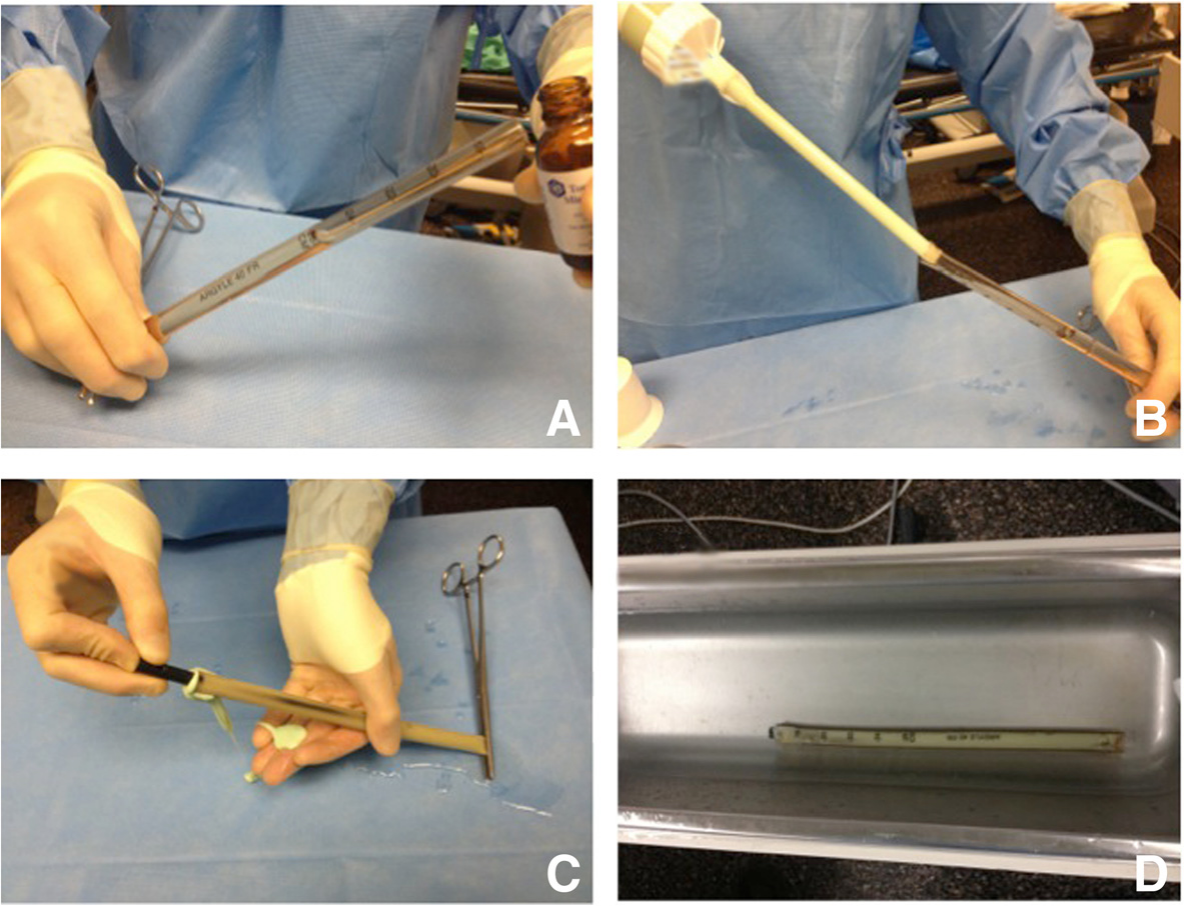
**Preparation of the antibiotic nail.** Loading the antibiotic nail: With one end of the chest tube closed with a Kocher **(A)**, sterile mineral oil is poured to coat the inner surface of the plastic tube. Using a cement gun to fill the chest tube mold **(B)**, the mineral oil is allowed to leak out under pressure. The carbon fiber nail is inserted into the antibiotic impregnated cement **(C)**, making sure to leave the threads exposed for the aiming arm. Once placement of the nail is adequate, the mold is placed in a cool sterile saline bath **(D)** to prevent the plastic from melting during the exothermic stage.

A 40 Fr chest tube is used as a mold. The chest tube is cut to the length of the carbon fiber nail. One end of the chest tube is cut longitudinally over 2 cm to facilitate peeling of the chest tube after the cement has hardened (Figure 2). A Kocher is used to seal off one extremity while the chest tube is lubricated with sterile mineral oil serving as a barrier between the cement and the plastic. Two grams of vancomycin powder (per 40 g cement) and 2.4 grams of Tobramycin (per 40 g cement) [14] are mixed together with 2 packs of low viscosity polymethyl methacrylate (PMMA) powder containing 0.5 grams of gentamicin (Palacos R + G, Zimmer, Warsaw, IN, U.S.A) and the liquid monomer is added (1 ampule of monomer per 40 g cement). The American Academy of Orthopaedic Surgeons (AAOS) issued guidelines for the use of cement spacers loaded with antibiotics in prosthetic joint infection [17]. Recommended doses range between 3 g to 8 g per 40 g of cement with the added comment that higher dose of antibiotics will compromise the structural characteristics of the construct. Vacuum mixing increases the eluting properties of the antibiotics when using Palacos cement. A cement gun is used to fill the chest tube. While the cement is still runny the interlocked carbon fiber nail is inserted into the tube. The proximal end of the nail should not be buried in cement in order to leave the threaded portion of the nail free of cement for attachment of the aiming arm (Figure 3).

**Figure 4:**
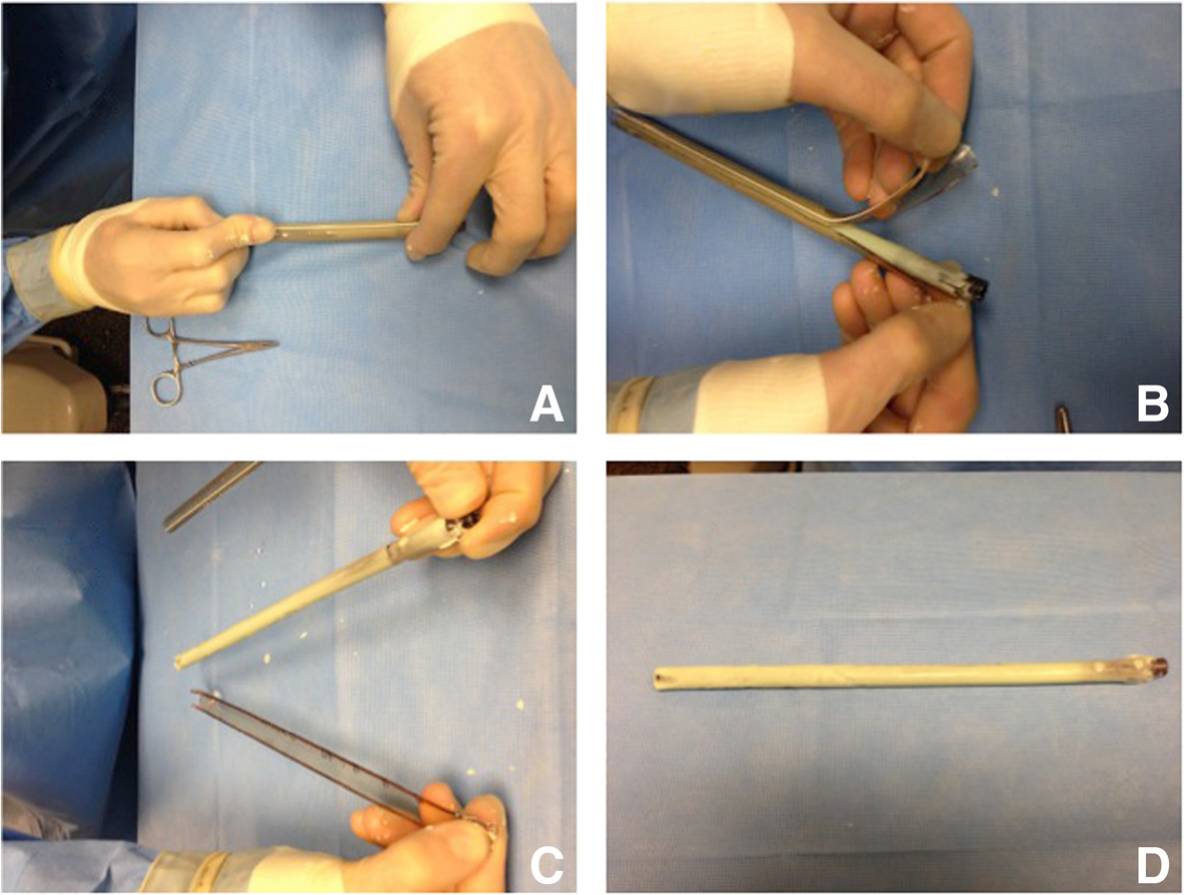
**Removal of the plastic mold from the cured antibiotic nail.** Nail completion: After the antibiotic impregnated cement has cured in the cooled saline bath, a scalpel is used to cut longitudinally along the length **(A)** starting at the two 2 cm cuts previously made. Grasping the side of the chest tube, pealing the plastic from the cement is effortless **(B and C)**. The nail is cured, smooth and ready to be attached to the aiming arm **(D)**.

A minute following insertion of the nail into the cement-filled chest tube and prior to the cement entering its exothermic phase, the construct is submerged into a cool sterile water basin. This step prevents heat accumulation and melting of the inner layer of the plastic. Following the cooling off period, the plastic tube is incised longitudinally with a scalpel on its two opposing sides. The plastic is easily peeled off from the cement rod (Figure 4).

**Figure 5:**
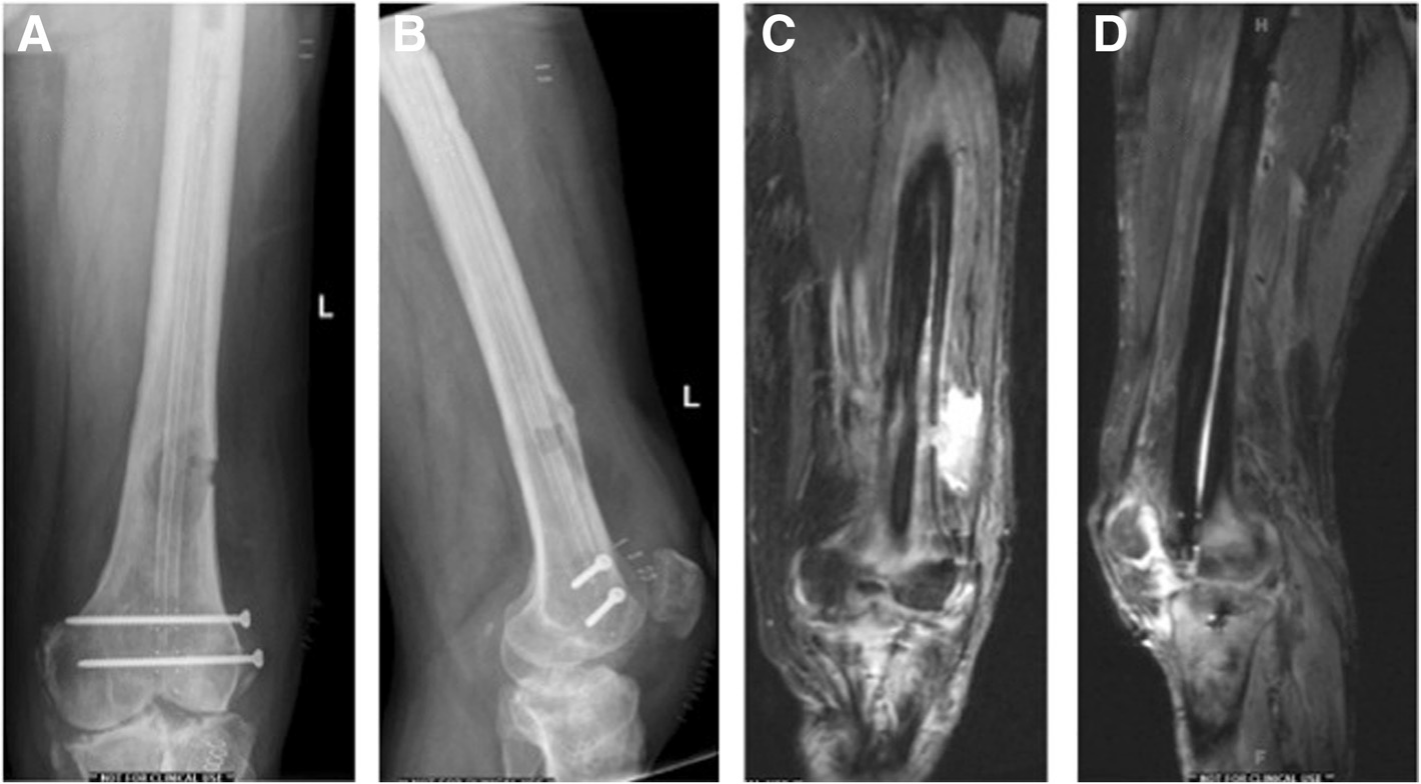
**Post-operative images. (A)** and **(B)** are AP and lateral postoperative X-rays of the distal femur demonstrating the radiolucent antibiotic impregnated cement carbon fiber interlocked nail with titanium screws. **(C)** and **(D)** are coronal and sagittal MRI STIR sequences demonstrating the absence of artifact This should allow for closer monitoring of the inflammation and disease progression.

The aiming arm attachment is assembled and introduced in a standard fashion. If required, interlocking screws are drilled and positioned through the cement mantle. Titanium screws are used for their MRI compatibility, post operative x-rays and MRI scan are obtained (Figure 5).

**Figure 6:**
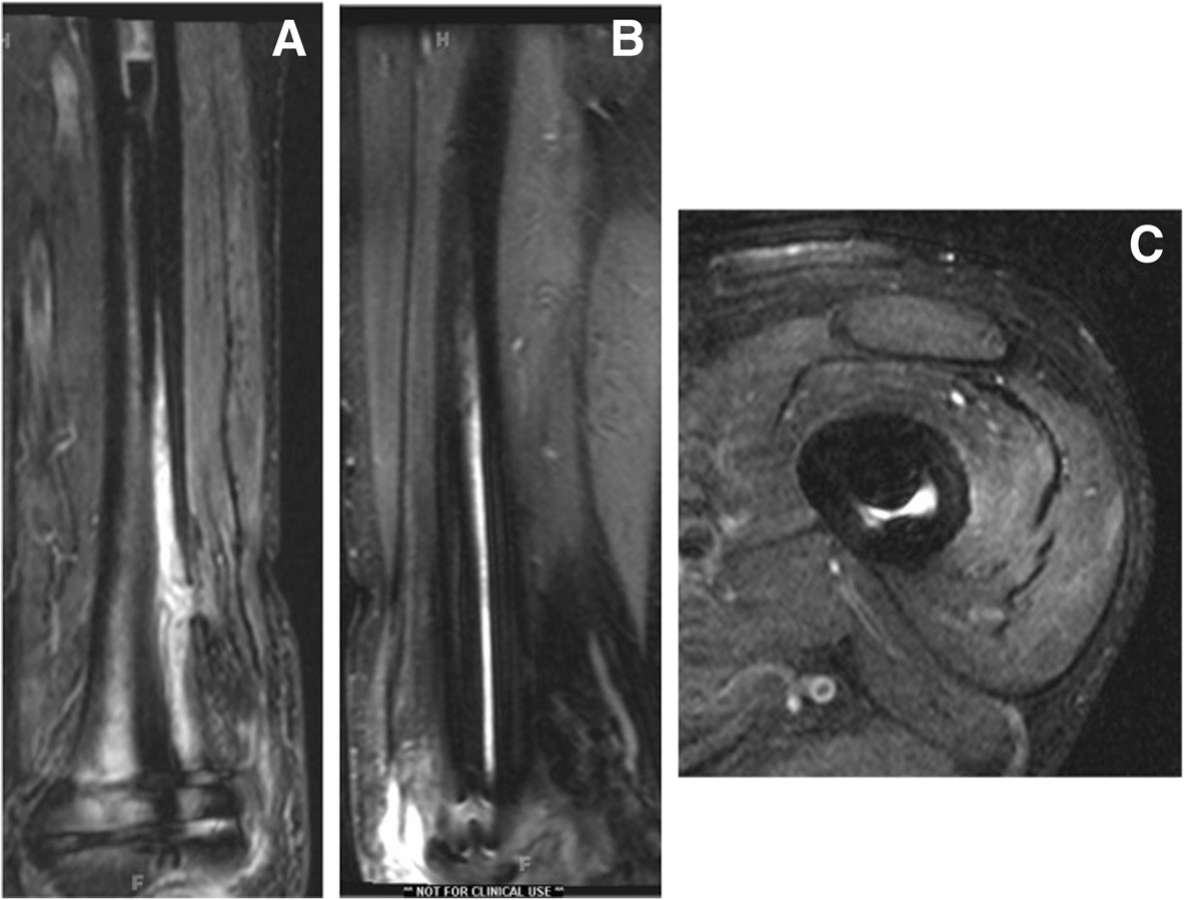
Follow up MRI scan with contrast at 8 weeks postoperative confirming overall reduction in size of osseous abscess and distal femoral enhancement on a coronal STIR (A), sagittal STIR (B) and axial STIR (C) of the distal femur.

The patient was followed up and reported significant improvement in his visual analogue pain scores from a previous 9/10 for thigh pain to 3/10 8 weeks post-operatively. The patient was able to get back to a full weight bearing status and walk several blocks with no thigh pain. His surgical scars healed uneventfully. An MRI was obtained 8 weeks following the antibiotic nail implantation looking for regression of the distal femoral infection and absence of inflammatory response to possible debris of mineral oil. The report confirmed significantly improved abscess extending through a cortical break in the distal femur with only minimal phlegmonous enhancement. The surrounding soft tissues of the distal thigh also demonstrated improved enhancement (Figure 6).

## Discussion

In this manuscript, we present a novel surgical technique for the fabrication of antibiotic nails using a radiolucent antibiotic carbon fiber interlocked devise. The authors are unaware of any previous publication of this technique. In our opinion, the addition of mineral oil and dipping of the antibiotic nail in a cold saline bath ensure a rapid, easy and reproducible method of fabrication. Interlocking the nail provides excellent rotational stability. The carbon fiber nail allows a follow up of the treatment response with limited metal artifact on MRI scan and visualization of the lesion on X-ray. This is especially valuable in immune-compromised patients where inflammatory markers are rarely reliable to monitor treatment response.

The use of interlocked intramedullary nails for intramedullary infections is not a novel concept and has previously been published for infected nonunion and delayed union of the tibia [18]. Carbon fiber nail for this indication is novel. We were unable to find any literature regarding carbon fiber nail use in long bone osteomyelitis despite the fact that carbon fiber technology use has been increasing over the last few years in Orthopaedics. Literature on carbon fiber implants in spine fusion confirms less artifact and improved evaluation of fusion compared with traditional implants [19-22]. It would logically follow that visualization would be improved in cases of bone infection.

Alternative implant options for treatment could have been a large wire coated in antibiotic cement. This method would certainly have decreased implant cost (the cost of the carbon fiber nail was $2,600) and perhaps limited the amount of exposed metallic surface for bacterial contamination. However, with our method of treatment, the patient is full weight bearing as opposed to partial weight bearing with a simple large pin and cement. In addition, the interlocking fixation minimizes the risk of migration of the construct proximally into the medullary canal and/or distally into the knee joint. Finally, although minimal, there would remain some MRI artifact created by the stainless steel pin. Given these disadvantages, this remains our preferred implant choice for this case scenario.

Our paper is by no means intended to recommend this methodology of treatment for all cases of long bone osteomyelitis or infected non-unions. The treatment of osteomyelitis is complex and needs to be individualized to each patient and problem [23]. We believe that this novel technique adds to the armamentarium to treat these difficult cases. Weakness of our manuscript includes the lack of long-term follow up. Our current research focus is to retrospectively evaluate a series of patients with long bone infection or infected non-unions treated using this technique. The use of mineral oil to coat the inner layer of the plastic tube may alter the elution properties of antibiotics loaded in the cement. In addition, mineral oil on the surface of the nail may create a localized inflammatory response (although the cured nail is copiously irrigated prior to its insertion). We could find no reports of this occurrence in the literature.

The presented use of antibiotic nail in general is ‘off- label’ according to current FDA indications. Our patients are informed during the shared decision making process. For research purposes our research division has contacted the FDA for suggestions on how to proceed with the prospective study of such implants.

### Unanswered questions

• Effects of mineral oil on antibiotics elution properties of the antibiotic nail

• Does the mineral oil cause a local inflammatory response?

• Is the interface between cement and carbon fiber adequate

• What are the elution properties of this construct and are the antibiotic eluted several weeks or months following nail insertion still active against causative organisms?

• Is MRI scanning a good tool to monitor response to treatment?

### Take-home-message

• Antibiotic coated intramedullary nails provide an excellent treatment modality in the treatment of certain cases of intramedullary long bone osteomyelitis. We describe a novel surgical technique using a carbon fiber interlocked antibiotic coated intramedullary nail.

• The addition of mineral oil in the chest tube and dipping of the construct in cold sterile saline facilitates the fabrication of the nail

• Utilization of a radiolucent interlocked implant improves MRI follow up of the infection site and X-ray visualization of the infected focus.

• This surgical technique solves many of the common issues with standard antibiotic nails and, unlike treatments using non-structural components to deliver the antibiotic, will provide support to the compromised bone. Clinical trials are needed to show the results of treatment with this novel technique.

## Competing interest

CM declares that he is the PI of a research grant funded by ‘Carbofix’ but did not receive any funds for the preparation of this manuscript.

## Authors’ contributions

CM had the idea of the surgical technique, conception of paper and editing. GC participated in the editing of the manuscript. NB helped in writing the manuscript and illustrations. HY did some editing on the manuscript. All authors read and approved the final manuscript.
